# The Inflammatory Burden in Heart Failure: A Cohort Study on Potential Biomarkers in Heart Failure With Reduced and Mildly Reduced Ejection Fraction

**DOI:** 10.7759/cureus.80159

**Published:** 2025-03-06

**Authors:** Emir Bećirović, Minela Bećirović, Kenana Ljuca, Mirza Babić, Amir Bećirović, Nadina Ljuca, Zarina Babić Jušić, Admir Abdić, Lemana Buljubašić, Emir Begagić

**Affiliations:** 1 Internal Medicine Clinic, University Clinical Centre Tuzla, Tuzla, BIH; 2 Gynecology and Obstetrics, University Clinical Centre Ljubljana, Ljubljana, SVN; 3 Department of Internal Medicine, Cantonal Hospital Bihać, Bihać, BIH; 4 School of Medicine, University of Tuzla, Tuzla, BIH; 5 Department of Surgery, Cantonal Hospital Bihać, Bihać, BIH; 6 Department of General Medicine, School of Medicine, University of Zenica, Zenica, BIH

**Keywords:** heart failure, inflammatory biomarkers, lmr, mlr, nlr, systemic inflammation

## Abstract

Background

Heart failure (HF) is characterized by impaired cardiac function. Based on left ventricular ejection fraction (LVEF), it is classified into HF with reduced ejection fraction (HFrEF), mildly reduced ejection fraction (HFmrEF), and preserved ejection fraction (HFpEF). Each phenotype has distinct pathophysiological mechanisms and clinical features. Recent findings indicate that systemic inflammation is a significant factor in the progression of heart failure. Inflammatory biomarkers, including neutrophil-to-lymphocyte ratio (NLR), monocyte-to-lymphocyte ratio (MLR), and lymphocyte-to-monocyte ratio (LMR), may serve as valuable tools for evaluating the inflammatory response in heart failure.

Materials and methods

This prospective observational study, which included 171 HF patients, was conducted from February 2022 to January 2023 at the Intensive Care Unit, University Clinical Centre Tuzla. Based on LVEF, patients were categorized into HFrEF, HFmrEF, and a control group (HFpEF). The study aimed to assess the prognostic value of NLR, MLR, and LMR in predicting major adverse cardiovascular events (MACE) and mortality over a 12-month follow-up period.

Results

NLR and MLR were significantly higher, while LMR was lower in both HFrEF and HFmrEF compared to controls, indicating a strong inflammatory response, particularly in HFrEF. NLR demonstrated a strong ability to distinguish between HF phenotypes. HFmrEF's markedly higher high-sensitivity troponin I (hsTroponin I) level suggested higher cardiac stress. MACE rates were similar across groups; mortality was significantly higher in HFrEF.

Conclusion

Inflammatory biomarkers NLR, MLR, LMR, and hsTroponin I could be crucial in assessing heart failure, particularly in patients with HFrEF and HFmrEF.

## Introduction

Heart failure (HF) is a complex clinical syndrome characterized by the heart’s inability to meet the body’s metabolic demands due to impaired pump function [1]. As a major public health concern, affecting approximately 64 million people worldwide, HF is a leading cause of hospitalization, with a five-year mortality rate exceeding 50% in advanced cases. This increasing prevalence could be attributed to an aging population and rising risk factors such as hypertension, diabetes, and coronary artery disease [2]. To enhance clinical outcomes, understanding the pathophysiological underpinnings of HF and identifying biomarkers that facilitate diagnosis, prognosis, and treatment strategies are paramount.

Left ventricular ejection fraction (LVEF) is the primary measure of left ventricular systolic function. It reflects the percentage of blood pumped out of the ventricle during systole compared to the total volume of blood in the ventricle at the end of diastole, typically ranging from 50% to 70% in healthy individuals [3]. HF is classified according to LVEF into three categories: heart failure with reduced ejection fraction (HFrEF; LVEF <40%), heart failure with mildly reduced ejection fraction (HFmrEF; LVEF 40-49%), and heart failure with preserved ejection fraction (HFpEF; LVEF ≥50%) [4]. Each group exhibits distinct pathophysiological mechanisms and clinical features. HFrEF is often linked to severe myocardial damage from conditions such as coronary artery disease, myocardial infarction, or chronic hypertension. The major structural abnormality of HFrEF is eccentric remodeling, followed by progressive ventricular dilatation and volume overload. In contrast, HFpEF is predominantly characterized by concentric remodeling accompanied by impaired myocardial relaxation and increased stiffness, resulting in pressure overload. It is frequently associated with conditions such as obesity, diabetes, and hypertension [4]. In clinical management, HFrEF often requires therapies targeting myocardial contractility and afterload reduction, while HFpEF treatment focuses more on managing comorbidities like hypertension and diabetes. HFmrEF may require a more comprehensive approach addressing systolic and diastolic dysfunctions. HFmrEF is still a relatively new classification since the exact pathophysiological mechanisms underlying HFmrEF are not yet fully understood, and ongoing research continues to explore its underlying mechanisms [5]. It was first introduced in the 2016 European Society of Cardiology (ESC) guidelines, and patients with HFmrEF may have both mild systolic dysfunction and diastolic dysfunction, encompassing characteristics of both HFrEF and HFpEF [6]. Comprehending these distinctions is essential for customizing therapeutic approaches. Recent research shows that systemic inflammation has a substantial role in the development and progression of HF, particularly in HFrEF [7]. The inflammatory response, which is induced by myocardial injury or hemodynamic stress, greatly contributes to poor cardiac remodeling, endothelial dysfunction, and disease progression. Inflammatory biomarkers and hematologic indicators have become widely available and inexpensive tools for reassessment and prognosis [7,8]. The neutrophil-to-lymphocyte ratio (NLR), monocyte-to-lymphocyte ratio (MLR), and lymphocyte-to-monocyte ratio (LMR) have all demonstrated potential in predicting the systemic inflammatory response associated with HF [9].

NLR is a simple biomarker that compares the absolute number of neutrophils to the absolute number of lymphocytes in the blood and it is calculated from a complete blood count. Neutrophils serve as the body's primary defense mechanism during the immune response. Elevated neutrophil counts are typically a sign of active inflammation and tissue injury. Lymphocytes are a key component of the adaptive immune response. Elevated NLR generally suggests a pro-inflammatory state, where neutrophil activity dominates, while a lower NLR indicates a stronger, more balanced immune response, where lymphocyte function is more prominent, which may reflect a healthier immune system and less inflammation. Increased NLR levels have been linked to poorer clinical outcomes, including elevated rates of hospitalization and mortality in HF patients [10].

Similarly, MLR is a biomarker that compares the absolute number of monocytes to the absolute number of lymphocytes in the blood. Monocytes help fight infections by phagocytosing pathogens and secreting inflammatory cytokines. They differentiate into macrophages and dendritic cells, which play key roles in inflammation and immune regulation. An elevated MLR suggests increased systemic inflammation as it reflects a higher number of monocytes relative to lymphocytes and it has been associated with detrimental cardiac remodeling and unfavorable outcomes. Conversely, LMR functions as an inverse marker of inflammation. This ratio compares the absolute number of lymphocytes to the absolute number of monocytes. An elevated LMR typically suggests a stronger adaptive immune response and a relatively lower inflammatory state, as lymphocytes are predominant. Low LMR indicates a shift toward a more inflammatory environment, as monocytes dominate, and it has been associated with poorer outcomes in cardiovascular illnesses [11]. High-sensitivity troponin I (hsTroponin I), a recognized indicator of myocardial damage, enhances these inflammatory ratios by detecting myocardial stress and injury, even in the absence of acute coronary syndromes (ACS) [12]. Given the increasing incidence of comorbid diseases in HF populations, such as hypertension, diabetes, and hyperlipidemia, the relationship between these risk factors and systemic inflammation is even more important. Identifying and incorporating inflammatory biomarkers into clinical practice could provide significant information about disease severity, guide therapeutic decisions, and enhance patient outcomes [13].

NLR, MLR, and LMR were chosen due to their potential ability to reflect different aspects of inflammation. Compared to traditional biomarkers like B-type natriuretic peptide (BNP) or N-terminal proBNP (NT-proBNP), these ratios could offer additional prognostic value in assessing systemic inflammation. BNP and NT-proBNP primarily indicate myocardial stretch, but they do not reflect the inflammatory milieu that contributes to disease progression in HF. HsTroponin I was selected as a complementary marker due to its role in detecting myocardial damage and stress. It enhances the inflammatory ratios by identifying patients with ongoing myocardial injury, even without ACS.

The research hypothesis is that inflammatory biomarkers (NLR, MLR, LMR) can provide valuable prognostic information for different HF phenotypes and contribute to optimizing treatment strategies. Clinically, these biomarkers may help refine risk stratification, guide therapy, and improve patient outcomes in HF management.

## Materials and methods

This study is a secondary analysis of a prospective observational study conducted from February 2022 until the end of January 2023 at the Intensive Care Unit of the Clinic for Internal Medicine, University Clinical Centre Tuzla, which included 171 consecutive patients diagnosed with HF. The available sample size was determined by the number of patients in the original dataset. Patients were categorized into three groups based on their LVEF: HFrEF, HFmrEF, and a control group of patients with HFpEF. HFpEF was chosen as the control group to focus on inflammation within heart failure, rather than comparing it to a non-heart failure population. This allows for a more relevant comparison of inflammatory biomarkers across heart failure subtypes, minimizing confounding variables related to non-cardiac factors.

The primary goal was to determine the prognostic value of NLR, MLR, and LMR in predicting major adverse cardiovascular events (MACE) and mortality across these LVEF categories. The inclusion criteria involved patients over 18 years of age diagnosed with heart failure through clinical presentation, echocardiographic findings, and elevated biomarkers, specifically hsTroponin I. Patients with severe valvular heart disease, acute infections, autoimmune disorders, active malignancies, recent trauma, end-stage kidney disease, and severe liver disease were excluded from the study. The dataset comprised demographic and medical history information. Clinical data were collected from electronic medical records, anamnesis, and physical examination.

Upon admission, patients received a thorough evaluation comprising an extensive clinical history, physical examination, and laboratory analysis. Blood samples were collected upon admission and underwent laboratory analysis for a complete blood count (CBC), serum hsTroponin I, creatinine, glucose, and C-reactive protein (CRP). The NLR, MLR, and LMR were calculated from CBC values. Laboratory studies were conducted via the Sysmex XN-1000 automated hematology analyzer (Sysmex Corporation, Kobe, Japan), with blood samples collected in K2EDTA tubes. Serum hsTroponin I concentrations were measured via the Beckman Coulter DxC 700 AU biochemical analyzer (Beckman Coulter Diagnostics, Nyon, Switzerland). Echocardiographic assessments were performed using the Vivid T8 ultrasound system (General Electric Medical Systems, Jiangsu, China). LVEF was quantified using the biplane Simpson method. Subsequent evaluations over a 12-month follow-up period included regular clinical exams and additional blood sampling to monitor biomarker changes and assess long-term outcomes.

Statistical analysis

The analyzed variables were tested for normality of distribution using the Kolmogorov-Smirnov test. Given that all variables exhibited deviations from a normal distribution, non-parametric tests were applied. Pearson’s χ² test was used for categorical variables, while the Kruskal-Wallis test was used for continuous variables. Categorical variables are presented as frequencies (No) and percentages, while continuous variables are presented as medians and interquartile ranges (IQR). For hemogram-derived ratios (HDRs) and inflammatory ratios where statistically significant differences between groups were observed, receiver operating characteristic (ROC) analysis was performed, and the area under the curve (AUC) index with 95% confidence intervals (CI) was determined, along with the cut-off value using the Gini coefficient of dispersion. Statistical significance was set at p < 0.05.

## Results

The demographic characteristics and medical history of the patients are outlined in Table 1.

**Table 1:** Baseline patients’ data HTA, Hypertension Arterial; DM T2, Diabetes Mellitus Type 2; HLP, Hyperlipidemia; MACE, Major Adverse Cardiovascular Events; HFrEF, Heart Failure with Reduced Ejection Fraction; HFmrEF, Heart Failure with Mildly Reduced Ejection Fraction; HFpEF, Heart Failure with Preserved Ejection Fraction

Variable		HFrEF (No = 30; 17.6%)	HFmrEF (No = 61; 35.9%)	Control (HFpEF) (No = 79; 46.5%)	p
		No (%)			
Age	<65	11 (36.7)	19 (31.1)	38 (48.1)	0.117
	≥65	19 (63.3)	42 (68.9)	41 (51.9)	
Sex	Male	21 (70.0)	36 (59.0)	46 (58.2)	0.507
	Female	9 (30.0)	25 (41.0)	33 (41.8)	
HTA		27 (90.0)	52 (85.2)	70 (88.6)	0.761
DM T2		12 (40.0)	24 (39.3)	35 (44.3)	0.821
HLP		21 (70.0)	46 (75.4)	57 (72.2)	0.842
Alcohol consumption		14 (46.7)	21 (34.4)	28 (35.4)	0.483
Tobacco consumption		16 (53.3)	29 (47.5)	40 (50.6)	0.863
MACE (12 months)		17 (56.7)	32 (52.5)	37 (46.8)	0.614
Mortality (12 months)		9 (30.0)	10 (16.4)	6 (7.6)	0.012

Among the 171 patients, the majority in each group were aged ≥65 (p = 0.117) and predominantly male (p = 0.507). Hypertension was prevalent in all groups, with no significant differences between HFrEF, HFmrEF, and controls. Type 2 diabetes, hyperlipidemia, alcohol consumption, and tobacco use were also commonly observed across the groups, with no significant differences in their prevalence between groups. The occurrence of MACE within 12 months was relatively similar, showing no significant difference (p = 0.614). However, 12-month mortality differed significantly between groups, with 30.0% of HFrEF patients, 16.4% of HFmrEF patients, and 7.6% of controls dying during this period (χ² test, p = 0.012). While MACE rates were comparable, mortality was significantly higher in the HFrEF group.

Table 2 presents the laboratory findings of the included patients.

**Table 2:** Laboratory findings among groups HDL, High-Density Lipoprotein; LDL, Low-Density Lipoprotein; CRP, C-Reactive Protein; hsTroponin I, High-Sensitivity Troponin I; HFrEF, Heart Failure with Reduced Ejection Fraction; HFmrEF, Heart Failure with Mildly Reduced Ejection Fraction; HFpEF, Heart Failure with Preserved Ejection Fraction

Variables	HFrEF	HFmrEF	Control (HFpEF)	p
	Median (IQR)			
Leukocytes (10^12^/L)	10.5 (8.0 - 14.4)	9.2 (7.7 - 11.3)	10.0 (7.7 - 11.8)	0.423
Erythrocytes (10^12^/L)	4.5 (4.1 - 4.8)	4.3 (4.0 - 4.9)	4.6 (4.0 - 4.9)	0.696
Hemoglobin (g/L)	135.0 (119.0 - 151.0)	135.0 (119.0 - 152.0)	138.0 (123.0 - 152.0)	0.737
Platelets (10^9^/L)	230.5 (190.0 - 299.0)	229.0 (182.0 - 277.0)	233.0 (180.0 - 260.0)	0.896
Monocytes (10^9^/L)	0.6 (0.5 - 0.9)	0.7 (0.5 - 0.9)	0.7 (0.5 - 0.9)	0.409
Basophils (10^9^/L)	0.0 (0.0 - 0.1)	0.0 (0.0 - 0.0)	0.0 (0.0 - 0.1)	0.393
Eosinophils (10^9^/L)	0.0 (0.0 - 0.1)	0.0 (0.0 - 0.1)	0.1 (0.0 - 0.1)	0.800
Lymphocytes (10^9^/L)	1.5 (1.1 - 2.0)	1.4 (1.1 - 1.8)	1.7 (1.2 - 2.3)	0.232
Neutrophils (10^9^/L)	7.3 (5.2 - 10.9)	6.5 (5.2 - 8.7)	6.9 (5.2 - 9.5)	0.800
Glucose (mmol/L)	8.9 (6.3 - 12.7)	7.3 (6.1 - 10.5)	7.6 (5.9 - 10.8)	0.789
Urea (mmol/L)	7.5 (5.3 - 11.7)	7.8 (5.9 - 10.4)	6.8 (5.4 - 10.3)	0.276
Creatinine (umol/L)	101.0 (81.0 - 126.0)	98.0 (79.0 - 146.0)	93.0 (72.0 - 112.0)	0.730
Triglycerides (mmol/L)	1.7 (1.2 - 2.1)	1.9 (1.4 - 2.3)	2.1 (1.2 - 3.0)	0.109
Cholesterol (mmol/L)	4.8 (4.0 - 6.2)	5.3 (4.6 - 6.6)	6.0 (4.7 - 7.1)	0.581
HDL (mmol/L)	1.3 (0.9 - 1.7)	1.2 (1.0 - 1.9)	1.2 (0.9 - 1.9)	0.065
LDL (mmol/L)	3.1 (2.5 - 3.5)	3.5 (2.9 - 4.0)	3.2 (2.4 - 4.1)	0.776
CRP (mg/L)	8.6 (3.0 - 21.0)	13.7 (2.9 - 58.2)	5.8 (1.9 - 20.0)	0.363
hsTroponin I (pg/mL)	673.3 (288.1 - 7069.1)	5756.1 (787.2 - 18542.8)	1202.4 (326.8 - 6007.2)	0.003

Most parameters, including hematological parameters, glucose, urea, creatinine, CRP, and various lipid levels, were similar across groups. However, hsTroponin I levels were significantly higher in the HFmrEF group.

Various inflammatory and immune-related indices across groups are shown in Table 3.

**Table 3:** Differences in hemogram-derived indices and inflammatory ratios among groups NLR, Neutrophil-to-Lymphocyte Ratio; dNLR, Derived Neutrophil-to-Lymphocyte Ratio; PLR, Platelet-to-Lymphocyte Ratio; MNR, Monocyte-to-Neutrophil Ratio; MLR, Monocyte-to-Lymphocyte Ratio; LMR, Lymphocyte-to-Monocyte Ratio; LCR, Lymphocyte-to-C-Reactive Protein Ratio; CRP/Ly, C-Reactive Protein-to-Lymphocyte Ratio; SII, Systemic Immune-Inflammation Index; AISI, Aggregate index of systemic inflammation; SIRI, Systemic Inflammation Response Index, High-Sensitivity Troponin I; HFrEF, Heart Failure with Reduced Ejection Fraction; HFmrEF, Heart Failure with Mildly Reduced Ejection Fraction; HFpEF, Heart Failure with Preserved Ejection Fraction

Variable	HFrEF	HFmrEF	Control (HFpEF)	p
	Median (IQR)			
NLR	4.3 (3.1 - 7.4)	4.0 (2.5 - 6.5)	2.5 (2.1 - 3.6)	<0.001
dNLR	2.7 (1.4 - 5.0)	2.1 (1.6 - 3.3)	1.9 (1.4 - 2.9)	0.522
PLR	148.0 (93.7 - 342.7)	145.2 (102.1 - 212.4)	119.0 (86.0 - 168.1)	0.122
MNR	0.1 (0.1 - 0.2)	0.1 (0.1 - 0.2)	0.1 (0.1 - 0.2)	0.753
MLR	0.5 (0.3 - 0.7)	0.5 (0.4 - 0.7)	0.4 (0.3 - 0.5)	0.005
LMR	2.2 (1.5 - 3.3)	2.0 (1.5 - 2.8)	2.7 (2.0 - 3.7)	0.005
LCR	0.1 (0.0 - 0.3)	0.0 (0.0 - 0.3)	0.1 (0.0 - 0.5)	0.086
CRP/Ly	10.8 (3.4 - 37.3)	22.3 (3.4 - 49.9)	7.1 (2.1 - 29.4)	0.053
SII	820.7 (509.7 - 2153.2)	960.3 (509.7 - 1449.0)	631.6 (400.6 - 1314.8)	0.169
AISI	597.6 (392.4 - 1394.2)	756.1 (349.6 - 1353.9)	449.7 (247.4 - 897.8)	0.131
SIRI	2.6 (1.5 - 5.7)	2.9 (2.0 - 6.1)	2.0 (1.3 - 4.2)	0.062

Significant differences were observed in inflammatory ratios such as NLR, MLR, and LMR, with HFrEF and HFmrEF showing higher levels of inflammation compared to controls. In particular, HFrEF has the highest median NLR (4.3), a significantly higher MLR (0.5), and a significantly lower LMR (2.2), indicating a higher level of systemic inflammation in the HFrEF group. In contrast, other indices like derived NLR (dNLR), platelet-to-lymphocyte ratio (PLR), and systemic immune-inflammation index (SII) do not show significant differences between the groups.

**Figure 1:**
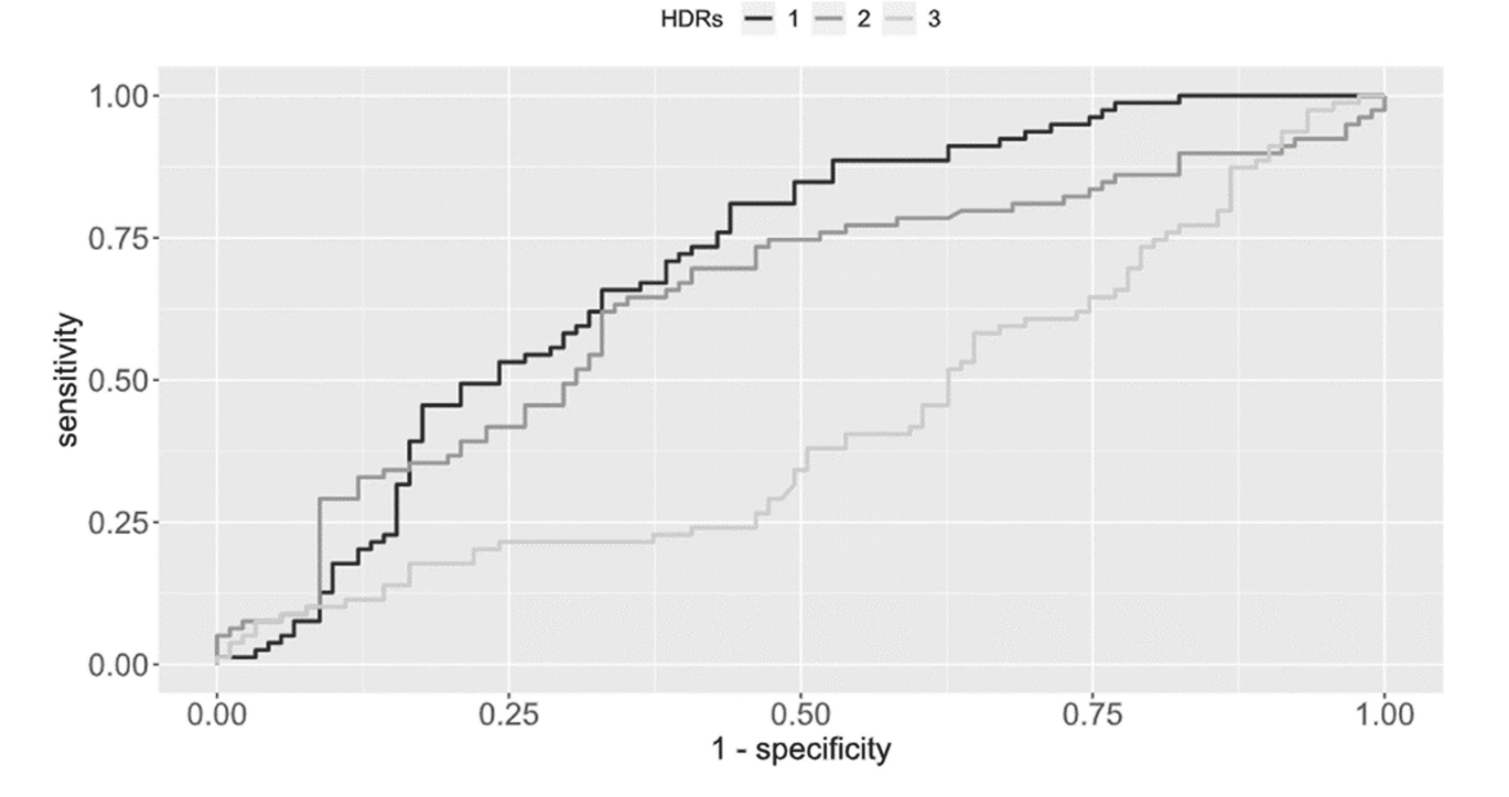
Receiver operating characteristic (ROC) analysis for discrimination of heart failure (ejection fraction < 40%) 1) Neutrophil-to-Lymphocyte Ratio (NLR) with Area Under Curve (AUC) = 0.740, 2) Monocyte-to-Lymphocyte Ratio (MLR) with AUC = 0.640, and 3) Lymphocyte-to-Monocyte Ratio (LMR) with AUC = 0.424. HDRs, hemogram-derived ratios

The inflammatory ratios that showed statistically significant differences between groups (p < 0.05) were further analyzed using ROC analysis (Figure 1).

For NLR, the AUC was 0.740 (95% CI: 0.636 - 0.793), indicating a strong ability to distinguish between the groups at a cut-off value of NLR ≥ 3.83 (p < 0.001). For MLR, the AUC was 0.640 (95% CI: 0.555 - 0.725), showing moderate discriminatory power at a cut-off value of 0.41 (p = 0.001). In contrast, LMR showed a lower AUC of 0.424 (95% CI: 0.337 - 0.511) at a cut-off of 0.71, which was not statistically significant (p = 0.088), suggesting poor ability to differentiate between groups.

## Discussion

Our study demonstrates a significant increase in hsTroponin I levels in HFmrEF, indicating ongoing myocardial damage in this group. NLR and MLR are significantly higher and LMR is considerably lower in HFrEF and HFmrEF compared to controls, revealing significant systemic inflammation in these groups. Inflammation is particularly pronounced in the HFrEF group. NLR exhibited a strong ability to differentiate between the groups.

Patients with HFmrEF exhibited markedly elevated levels of hsTroponin I, emphasizing its role as a sensitive marker of myocardial stress and injury [14]. This elevation may reflect subclinical ischemic events and increased wall stress due to impaired yet preserved systolic function [15-17]. Our study further confirms the theory that HFmrEF seems to represent a transitional heart failure phenotype sharing inflammatory and structural remodeling features with HFrEF, though with less severe myocardial damage [18].

The NLR was considerably greater in patients with HFrEF than in those with HFmrEF or in the control group, indicating that it has the potential to be a cost-effective risk classification tool [19]. The increase in NLR corresponds to a heightened systemic inflammatory response causing cardiac damage. In HFmrEF, inflammation persists but is less severe, as indicated by intermediate NLR levels when compared to HFrEF and controls [20]. This biomarker is linked to adverse clinical outcomes, including increased rates of hospitalization and mortality [21]. Mortality was highest in the HFrEF group that demonstrated the highest NLR levels, suggesting its potential role as a marker of negative outcomes. Elevated NLR levels demonstrate a strong discriminatory ability to identify HF patients. The cut-off value is associated with the severity of systemic inflammation. Identifying patients with NLR levels over this threshold can facilitate proactive early interventions to alleviate illness development. Given the role of inflammation in HF progression, potential therapeutic strategies targeting inflammatory pathways, such as anti-inflammatory agents or cytokine modulation, may improve clinical outcomes, but future studies are needed to explore the potential efficacy of these interventions.

Moreover, the HFrEF group showed a significantly higher MLR, suggesting monocyte activation and its role in promoting fibrosis and myocardium remodeling, which are central processes to HF pathophysiology. Although less pronounced than in HFrEF, high MLR in HFmrEF emphasizes persistent low-grade inflammation, which accelerates the disease progression [22,23].

In our study, the LMR was significantly reduced in both HFrEF and HFmrEF when compared to controls. This reduction further confirms chronic inflammation and immune dysregulation [24]. In HFrEF, more pronounced lymphopenia leading to a very low LMR suggests advanced systemic inflammation. In HFmrEF, decreased LMR indicates moderate inflammation, reflecting its intermediate clinical phenotype between HFrEF and controls [25]. Reduction in LMR is associated with poorer outcomes in cardiovascular diseases, highlighting its prognostic significance in HF [26].

Elevated NLR levels demonstrate a strong discriminatory ability to identify HF patients, confirming their prognostic importance [27]. The cut-off value is associated with the severity of systemic inflammation, a significant contributor to negative outcomes in heart failure, such as increased hospitalization and mortality rates. Identifying patients with NLR levels over this threshold can facilitate proactive early interventions to alleviate illness development [28]. Likewise, MLR demonstrated statistical significance, endorsing its clinical relevance [29]. The results, coupled with the death rates of 30.0% in HFrEF, 16.4% in HFmrEF, and 7.6% in controls, highlight the imperative of incorporating these biomarkers into therapeutic practices to improve outcomes. These results highlight the importance of integrating inflammatory indicators into therapeutic practice; however, further validation in larger cohorts is necessary to confirm their clinical utility and impact on patient outcomes. NLR, MLR, and LMR measurements are important tools for risk classification and treatment decision-making since they are simple and cost-effective [30].

Limitations

This study provides valuable insights but has several limitations. As a secondary analysis, it relies on pre-existing data, which may limit the ability to control for confounding variables or introduce biases based on the original study design. Additionally, as a single-center study, there is potential for selection bias, which may reduce the generalizability of our findings. Larger, multicenter studies with more diverse populations are needed to confirm these results. Residual confounding from unmeasured variables, such as dietary factors, physical activity, genetic predispositions, or underlying inflammatory conditions, may have influenced inflammatory marker levels. The cross-sectional design further restricts our ability to assess temporal changes in inflammatory markers or evaluate the impact of heart failure treatment on these biomarkers. Future longitudinal studies are needed to address these gaps.

## Conclusions

Our study reveals the potential value of NLR, MLR, LMR, and hsTroponin I as cost-effective and sensitive biomarkers in understanding and predicting heart failure, particularly in HFrEF and HFmrEF patients. These biomarkers may be integrated into clinical workflows for risk stratification, monitoring disease progression, and guiding anti-inflammatory therapies. While the pathways linking systemic inflammation to myocardial injury, particularly during the transition from HFmrEF to HFrEF, require further investigation, these markers could play an important role in managing heart failure in the future. However, due to the cross-sectional design and single-center nature of the study, caution should be exercised, and external validation is necessary. Future research should focus on longitudinal studies, interventional trials targeting inflammation, and studies exploring immune cell involvement.
